# Behavioral Theories and Motivational Features Underlying eHealth Interventions for Adolescent Antiretroviral Adherence: Systematic Review

**DOI:** 10.2196/25129

**Published:** 2021-12-10

**Authors:** Alemitu Mequanint Bezabih, Kathrin Gerling, Workeabeba Abebe, Vero Vanden Abeele

**Affiliations:** 1 Department of Computer Science, e-Media Research Lab Katholieke Universiteit Leuven Leuven Belgium; 2 Department of Pediatrics and Child Health Addis Ababa University Addis Ababa Ethiopia

**Keywords:** HIV, adolescents, ART adherence, eHealth, health theories, behavior change techniques, motivational design principles

## Abstract

**Background:**

eHealth systems provide new opportunities for the delivery of antiretroviral therapy (ART) adherence interventions for adolescents. They may be more effective if grounded in health behavior theories and behavior change techniques (BCTs). Prior reviews have examined the effectiveness, feasibility, and acceptability of these eHealth systems. However, studies have not systematically explored the use of health behavior theories and BCTs in the design of these applications.

**Objective:**

The purpose of this review was to explore whether health behavior theories and BCTs were considered to ground designs of eHealth systems supporting adolescents’ (10-24 years) ART adherence. More specifically, we examined which specific theories and BCTs were applied, and how these BCTs were implemented as design features. Additionally, we investigated the quality and effect of eHealth systems.

**Methods:**

A systematic search was performed on IEEE Xplore, ACM, ScienceDirect, PubMed, Scopus, and Web of Science databases from 2000 to 2020. Theory use and BCTs were coded using the Theory Coding Scheme and the Behavior Change Technique Taxonomy version 1 (BCTTv1), respectively. Design features were identified using the lenses of motivational design for mobile health (mHealth). The number of BCTs and design features for each eHealth system and their prevalence across all systems were assessed.

**Results:**

This review identified 16 eHealth systems aiming to support ART adherence among adolescents. System types include SMS text message reminders (n=6), phone call reminders (n=3), combined SMS text message and phone call reminders (n=1), electronic adherence monitoring devices (n=3), smartphone apps (n=1), smartphone serious games (n=1), gamified smartphone apps (n=1), leveraging existing social media (n=2), web-based applications (n=1), videoconferencing (n=1), and desktop applications (n=1). Nine were grounded in theory, of which 3 used theories extensively. The impact of adolescent developmental changes on ART adherence was not made explicit. A total of 42 different BCTs and 24 motivational design features were used across systems. Ten systems reported positive effects on 1 or more outcomes; however, of these ten systems, only 3 reported exclusively positive effects on all the outcomes they measured. As much as 6 out of 16 reported purely no effect in all the outcomes measured.

**Conclusions:**

Basic applications (SMS text messaging and phone calls) were most frequent, although more advanced systems such as mobile apps and games are also emerging. This review indicated gaps in the use of theory and BCTs, and particularly the impact of developmental changes on ART adherence was not adequately considered. Together with adopting a developmental orientation, future eHealth systems should effectively leverage health theories and consider developing more advanced systems that open the door to using BCTs more comprehensively. Overall, the impact of eHealth systems on adolescent ART adherence and its mediators is promising, but conclusive evidence on effect still needs to be provided.

## Introduction

### Background

HIV disproportionately affects adolescents worldwide (an extended definition covering ages 10-24 [1] is used here). AIDS is the first cause of death among adolescents in Africa and the second worldwide [2]. There is no cure for HIV yet. However, antiretroviral therapy (ART) is an effective measure to control HIV if properly adhered to. Unfortunately, suboptimal ART adherence is common among adolescents with HIV. While there is an overall improvement in other age groups in the epidemic control of HIV, when compared with other age categories [3], adolescents are characterized by (1) higher treatment dropout rate [4,5], (2) lower viral suppression achievement [6], (3) rising AIDS-related illnesses [5], and (4) a smaller decrease in AIDS-related deaths.

While adolescents share several barriers to ART adherence with adults [7], there are also challenges unique to adolescents that further complicate adherence [8-12]. Some of these challenges emerge from the unique developmental changes associated with adolescence, including biological, cognitive, and psychosocial changes [13-19]. Adolescence is a period of cognitive maturation; however, this is a gradual process, and (younger) adolescents are still limited in formal and hypothetical thinking [17]. In periods of stress, even older adolescents may regress to simplistic preformal reasoning. Consequently, they may not foresee the long-term importance of ART adherence and underestimate the severity of the HIV condition and the susceptibility of facing nonadherence consequences [20]. This in turn can lead to risk-taking behaviors. Additionally, adolescence is a period of becoming autonomous in which control over their own lives is paramount [21]. Therefore, threats to personal agency, for example, impositions from health professionals, requesting to comply with ART, are likely met with psychological reactance, an aversive response, possibly resulting in nonadherence. Adolescence is also a developmental period characterized by an orientation toward the peer group and the need to conform [22]. Because of the need to maintain appearances and fit in with their peers, and possibly engage in sexual relations [8], adolescents may choose to hide or even completely deny having HIV, especially if the disease is asymptomatic. As an unfortunate consequence, nonadherence to ART and death rates among adolescents living with HIV are disconcerting.

### Prior Work

In the last decade, the availability of eHealth systems has dramatically increased [23]. eHealth refers to health services, information, and support that are delivered or enhanced through web-based technologies and related software applications [24], including SMS text messaging, web-based applications, social media, mobile apps, and games. eHealth interventions are also increasingly being used to improve ART adherence, and integrated into HIV self-management and service delivery [25-28]. Overall, eHealth is considered a promising approach to deliver effective interventions for ART treatment, for both adult [29-32] and adolescent groups [27,33-35].

ART adherence is a complex health behavior determined by multiple sociobehavioral factors, and for adolescents, further complicated by unique developmental changes at the biological, social, and psychological level. Prior reviews of eHealth ART adherence systems for adolescents have examined the effectiveness, feasibility, and acceptability [33-36] and found that such systems have generally encouraging impact. Similar findings have also been synthesized for other chronic health conditions [34,37-39]. However, to the best of the authors’ knowledge, no reviews have systematically explored the extent to which health behavior theories ground the intervention, guide the selection of behavior change techniques (BCTs), or inform the design features in the app itself. This is surprising, as several studies suggest that grounding the design of eHealth systems in theory is associated with increased effectiveness [40-42]. Specific to HIV, recent systematic reviews also revealed that, for adults, designing eHealth systems based on behavioral theory is associated with efficacy in improving adherence to HIV medication [30].

Health behavior theories may provide a comprehensive understanding of adolescent’s adherence behavior and its determinant factors [43]. A number of theories are already applied to model HIV medication adherence [44], including the Health Belief Model [45], Information–Motivation–Behavioral Skills Model [46,47], Social Cognitive Theory [48], Theory of Reasoned Action [49], Theory of Planned Behavior [50], and Transtheoretical Model [51]. Therefore, health behavior theories can provide insights on achieving behavior change, and hence could support the design of eHealth systems that specifically consider adolescent adherence behavior.

Derived from the aforementioned theories and models, several behavior change strategies exist that may influence, motivate, or persuade people to adhere to healthy behaviors. These strategies are organized into taxonomies such as the Behavior Change Technique Taxonomy version 1 (BCTTv1) of Michie et al [52] or Cialdini’s influence techniques [53]. Systematic reviews also revealed that using such specific techniques facilitate behavior change, and are associated with efficacy in improving adherence to HIV medication [30]. Note that while these frameworks are applied to clinical practice, they are still technology agnostic. They apply to human-delivered interventions, first and foremost.

Hence, for eHealth interventions, such BCTs still need translation into designed features of a delivery platform. Also here, frameworks exist to guide researchers, such as Fogg’s persuasive principles [54,55], persuasive system design principles of Oinas-Kukkonen and Harjumaa [56], lenses of motivational design for mHealth features by Geuens et al [57], or taxonomies of gamification elements [58-60]. Different from the BCTs, these necessitate and describe features of a technology-enabled intervention. Despite their applied nature, here too, research has shown the need for design considerations to be based on health behavior change theories [40,42,61].

**Figure 1:**
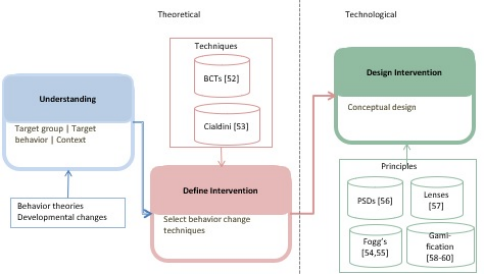
A stepwise design process of e-Health behavioral interventions. BCT: behavior change technique; PSD: Persuasive Systems Design.

In sum, designing an eHealth intervention implies a 3-stage process: (1) understanding health behaviors through insight from appropriate *theories*, (2) defining appropriate BCTs as elements of the intervention, and (3) a translation into designed *features* for a chosen eHealth platform [42,57,62]. An illustration of this pipeline is given in Figure 1. Ideally, step 3 (designing features) is proceeded by steps 1 (understanding through theory) and 2 (specifying BCTs) [57]. However, this is not a mandate and researchers/app designers may jump to step 3 directly [42]. Considering this design pipeline perspective, it is therefore interesting to investigate whether theories matter for quality and impact of eHealth interventions for ART adherence of adolescents as well [42].

### Research Objectives

The purpose of this systematic review is to assess the extent to which studies implement the different steps of this pipeline (health behavior *theory* > *BCT* > designed *features*) and how this is related to the quality and impact of eHealth systems to promote adolescents’ ART adherence. Therefore, we explore the research questions (RQs) in Textbox 1.

**Research questions (RQs).**

- RQ1. Are eHealth interventions addressing antiretroviral therapy (ART) adherence among adolescents with HIV grounded in health behavior theory?
  Which theories are used?
  How often were theories used?
  Do theories used specifically address developmental changes related to adolescence?
- RQ2. Do the eHealth interventions addressing ART adherence among adolescents rely on behavior change techniques (BCTs)?
  Which BCTs are used?
  How many BCTs are used by interventions?
  Are BCTs linked to behavior change theories?
- RQ3. How are eHealth interventions addressing ART adherence among adolescents implementing the BCTs as design features?
  What platforms are chosen?
  Which features are designed?
  How many design features are used in the different platforms?
- RQ4. What are the quality and impact of the eHealth interventions and how do they relate to grounding in theories related to health behavior and behavior change?
  What is the evidence quality of the interventions?
  What is the impact of the interventions?
  What is the relation between grounding in theory and impact of the interventions?

## Methods

### Overview

The process of formulating the RQs and the search strategy was guided by the PICO(S) (Patient/Population/Problem, Intervention, Comparison, Outcome, Study design) concept of Cochrane Collaboration [63]. We used the PRIMA guidelines as a basis for conducting and reporting this systematic review [64].

### Search Strategy

The search query was crafted as a combination of the PICO text words and then applied on the databases, limited to metadata fields search (title, abstract, and keyword). We restricted the search on eHealth interventions pertaining to ART adherence among adolescents, excluding those regarding other HIV care services such as HIV testing. Search terms were structured as per the syntax of each database (Multimedia Appendix 1). The entire search query was refined via several tests and peer reviews. To expand the search into an interdisciplinary space, electronic databases relevant to technology and medical fields (IEEE Xplore, ACM, ScienceDirect, PubMed, Scopus, and Web of Science) were searched on April 25, 2019, including all papers up to this date. A secondary search was conducted on all databases on January 22, 2021, to check for new relevant citations. The search sting was formulated as follows:

[HIV OR “Human Immunodeficiency Virus” OR “HIV/AIDS” OR “Acquired Immunodeficiency Syndrome” OR “HIV-positive” OR “HIV+” OR “living with HIV”] AND [adolescent OR teen* OR young OR youth] AND [ARV OR antiretroviral OR “Antiretroviral Therapy” OR “HIV treatment” OR “HIV care” ] AND [“eHealth” OR “e-health” OR “electronic health” OR “digital health” OR telemedicine OR “tele-medicine” OR technology OR “computer-based” OR “web” OR “web-based” OR Internet OR online OR “social media” OR “social networking” OR “mHealth” OR “m-health” OR “mobile health” OR “mobile phone” OR “cell phone” OR “cellular phone” OR smartphone OR “text message” OR SMS OR “short message service” OR “app” OR “application” OR game OR videogame OR gamif* OR “play”] AND [adherence OR attrition OR dropout OR drop-out OR completers OR “lost to follow-up” OR withdrawal OR nonresponse OR non-response OR “completion” OR “did not complete” OR retention OR loss OR compliance OR concordance]

### Eligibility Criteria

This review focused on eHealth interventions designed for adolescents to support ART adherence. We developed and applied the inclusion/exclusion criteria listed in Textbox 2.

**Inclusion and exclusion criteria.**

**Inclusion Criteria**

- eHealth interventions designed for adolescents (age average between 10 and 24 years) to improve HIV medication adherence.
- Studies clearly describing the intervention content and characteristics.
- Interventions empirically evaluating antiretroviral therapy (ART) adherence, measured as one of the following:
  Primary adherence behavior–related outcomes (eg, change in knowledge and attitude about HIV and ART, self-efficacy in taking ART medication, social support).
  ART adherence (directly measured):
    Objective measure (eg, using real-time electronic adherence monitoring, pill count).
    Subjective measure (eg, self-report, caregiver report).
  Biological outcomes (eg, CD4 count, viral load suppression).
- Peer-reviewed articles published in English (journal or conference).

**Exclusion Criteria**

- Interventions designed for other age groups or health professionals (or mixed but no age subgroup analysis).
- Abstracts, reviews, protocols (however, we kept papers describing an already included eHealth system to get extra information about the intervention), ongoing works, books, book chapters.
- Interventions that only evaluated user experience, acceptability, or feasibility but not ART adherence.
- Interventions that focused on other HIV care services such as reducing risky behaviors, promoting HIV testing, or pretreatment care.

### Screening and Inclusion

**Figure 2:**
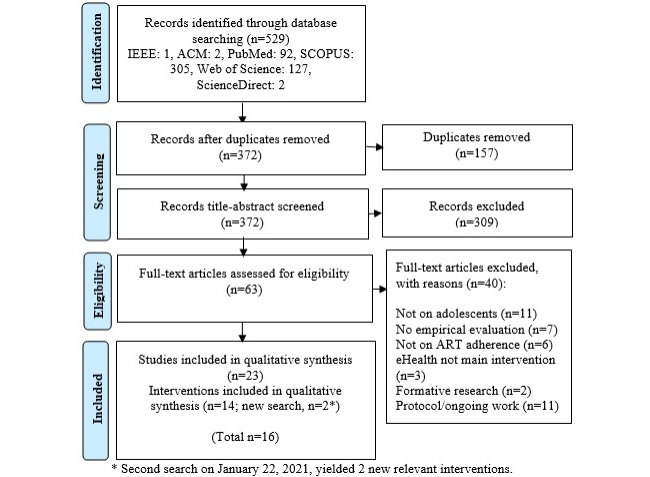
Screening and inclusion process.

A multistep screening process (Figure 2) was applied to get to the final included interventions. As a first step, 529 papers were obtained, and duplicates (n=157) were removed. Next, 372 unique papers were screened by applying the criteria in Textbox 2 on both title and abstract. After this screening, 63 papers remained. In case of doubt on title/abstract information, papers were simply added to the next phase. In the fourth step, the 63 full papers were read and screened against the inclusion/exclusion criteria, resulting in further removal of 40 papers, eventually retaining 23 papers describing 14 different interventions. When available, various papers on an intervention (protocol, feasibility study, user experience study, pilot test, and randomized controlled trial [RCT]) were all kept to the end for further reference of intervention details. In April 2021, a second database search was performed to capture any new studies, resulting in 165 new unique citations. Following the same screening process, 11 papers were screened (after excluding 154) via title/abstract screening. Full-text reading of these papers resulted in 2 papers describing 2 eHealth systems for final inclusion. Totally, out of 694 papers from both searches, 16 different eHealth apps were included and analyzed.

### Data Extraction and Analysis

We coded relevant characteristics including intervention name, publication year, country, delivery technology, study design, population, sample size, follow-up duration, intervention summary, theory use and justification of theory selection, consideration of developmental changes related to adolescence, outcomes, and effectiveness.

### Coding of Behavior Theories, Behavior Change Techniques, and Designed Features

#### Behavior Change Theories

We used the Theory Coding Scheme (TCS) developed by Michie and Prestwich [65] to describe the theory basis of each intervention and to assess to what extent theories were utilized. This checklist has 19 items to verify whether an intervention mentions a theory or model, to what extent a theory or model is used in designing intervention features, and whether the theory is tested in pre/poststudy. Item 1 verifies whether a particular theory is mentioned in the studies, whereas items 2 and 4-11 measure to what extent the theory is used during designing interventions. Item 3 checks whether more than 1 theory is applied. Item 12a verifies whether the impact on adherence mediators was measured. The remaining items were left uncoded as they deal with reliability of methods in measuring and testing theory, and theory refinement, which is beyond the scope of this study. Each item was coded as 1 if applied and 0 if not present. As a last step and composite measure, the included eHealth systems were graded on the extent of theory usage as no, low, medium, or high usage. Studies were labeled as follows: “no,” if there was no mention of theory, or they mentioned a theory but there was no grounding of the intervention on it (TCS item 1); “low,” if it was explicitly stated that theory concepts were used to design the intervention (1 or more of the TCS items 2, 5, 8, and 11); “medium,” if all intervention techniques originated from theory concepts (TCS item 7 or 9); “high,” when all theory concepts were addressed, or used to select participants or tailor interventions (1 or more of TCS items 4, 6, and 10).

#### Consideration of Developmental Changes

We investigated studies for the presence of discussions concerning how developmental theory concepts influence ART adherence among adolescents. This examination was particularly inspired by TCS item 1 which says “Theory/model of behavior mentioned—Models/theories that specify relations among variables, in order to explain or predict behavior are mentioned, even if the intervention is not based on this theory” [65]. Hence, we examined the presence of explicit discussions on how developmental changes (biological, social, psychological) would “explain or predict” (to use the exact words) ART adherence among adolescents.

#### Behavior Change Techniques

For coding BCTs from intervention descriptions, BCTTv1 [52] was used. BCTTv1 is a well-established taxonomy with an extensive list of theoretical methods of behavior change, containing 93 BCTs grouped into 16 clusters. It has been widely applied to specify intervention techniques in various behavioral domains such as physical activity [66], alcohol use [67], and medication adherence [68]; as well as to identify the presence of BCTs in existing interventions [41,62]. To examine the use of BCTs in interventions*,* the prevalence of individual BCT across all interventions was calculated. Similarly, we also calculated the total number of BCTs per intervention. Considering the complexity of coding BCTs, coders completed a certified online training on BCT taxonomy (BCTTv1) [69].

#### Designed Features

Because BCTs are only descriptions of behavior change from a psychological perspective, in other words they are technology agnostic, we additionally coded the designed features, using the lenses of motivational design for mHealth developed by Geuens et al [57]. Geuens et al [57] explained how theoretical concepts of behavior change can be translated into design principles, by including also implementation instantiations of these principles through examples of mobile app features. These lenses of motivational designs provide implementation-level descriptions of design principles, also encompassing persuasive principles of [54-56]. The prevalence of individual design features among systems and the number of features per each system are calculated.

#### Impact

Because interventions differ substantially in measuring their impact, *each outcome measure* was coded (mediators, adherence, or health outcomes) as positive effect (+), no effect (0), or negative effect (–). Hence, we did not exclude any outcome measures or limit ourselves to direct measures of adherence such as medication intake or viral load. This inclusive approach is justified by the Information–Motivation–Behavioral Skills Model [47] of ART adherence, as it accommodates a broad range of outcome measures [47,70], and is widely adopted in ART adherence research [71-73]. This model conceptualizes ART adherence as a function of 3 mediators: information about HIV and ART, motivation to take medication, and behavioral skills required for taking medication. An increase in any of these constructs is theorized to result in improved adherence behavior which in turn produces favorable health outcomes.

### Methodological Quality Assessment

Finally, we evaluated the strength of evidence for the methodological quality of included interventions with respect to the review questions, using the quality assessment method used in Johnson et al [74], with a minor modification: we discerned pre/posttest studies from single-subject and case studies (Table 1). This method was first used to assess the quality of evidence for the impact of computer games and serious games on learning [75,76], and later for their impact on health and well-being [74]. It has 5 criteria against which every included intervention is scored from 1 to 3. Adding up each of the 5 marks gives a possible maximum score of 15. A subsample of interventions (4/15, 26.67%) was coded independently by 2 coders (first and last authors). Interrater reliability was calculated using intraclass correlation coefficient with 2-way mixed effects and absolute agreement. The score was 0.89, showing a good agreement between the 2 coders. The quality measure here refers to the quality of clinical validation test (study design, eg, RCT, pre/post; sample size), not the quality in terms of design and development of eHealth systems (ie, integration of theory, BCTs, design elements).

**Table 1:** Evidence quality assessment method.

Criterion	Min score	Max score
How appropriate is the research design for addressing the question, or subquestions of this review: randomized controlled trails (3), quasi-experimental study (2.5), pre/posttest design (2), case study, single subject-experimental design (1)?	1	3
How appropriate are the methods and analysis?	1	3
How generalizable are the findings of the study to the target population with respect to the size and representativeness of sample?	1	3
How relevant is the particular focus of the study (including conceptual focus, context, sample, and measures) for addressing the question or subquestions of this review?	1	3
To what extent can the study findings be trusted in answering the study question(s)?	1	3

## Results

### Overview

Of the 16 included systems, most were built in the United States (n=12), 2 in Nigeria, 1 in Uganda, and 1 in Argentina. Some studies (n=5) intentionally included participants with poor adherence performance. Half of the systems employed pre–post designs, whereas the other half used RCTs for clinical validation. Table 2 shows the list of included studies, and the summary of interventions is provided in Multimedia Appendix 2.

**Table 2:** Included studies.

No.	Intervention studies^a^	References
1	Whiteley et al	[77,78]
2	Tanner et al	[79,80]
3	Stankievich et al	[81]
4	Spratt et al	[82]
5	Shegog et al	[83]
6	Belzer et al	[84-86]
7	Saberi et al	[87]
8	Linnemayr et al	[88,89]
9	Puccio et al	[90]
10	Naar-King et al	[91,92]
11	Hightow-Weidman et al	[93]
12	Dowshen et al	[94,95]
13	Garofalo et al	[96]
14	Dworkin et al	[97-99]
15	Dulli et al	[100]
16	Abiodun et al	[101]

^a^For interventions with multiple studies, only the first author of one of the papers is used.

### RQ1: Are eHealth Interventions Grounded in Behavior Theory?

#### Which Theories Were Commonly Applied to Inform Behavior Change?

A total of 10 different theories were mentioned (Table 3), of which 3 theories appeared commonly, namely, Social Cognitive Theory, the Information–Motivation–Behavioral Skills Model, and Motivational Interviewing. The complete theory coding sheet of included studies using the TCS is provided in Multimedia Appendix 3.

**Table 3:** Theories used by the included interventions.

Theory	Frequency	Interventions
Social Cognitive Theory	4	Tanner et al [79,80], Shegog et al [83], Hightow-Weidman et al [93], Garofalo et al [96]
Information–Motivation–Behavioral Skills Model	3	Whiteley et al [77,78], Linnemayr et al [88,89], Dworkin et al [97-99]
Motivational Interviewing	3	Spratt et al [82], Shegog et al [83], Naar-King et al [91,92]
Fogg Behavior Model	1	Hightow-Weidman et al [93]
Empowerment Theory	1	Tanner et al [79,80]
Transtheoretical Model	1	Spratt et al [82]
Stress, Appraisal, and Coping Theory	1	Belzer et al [84-86]
Narrative Communication (Storytelling)	1	Hightow-Weidman et al [93]
Ecological Momentary Intervention	1	Dowshen et al [94,95]
Social Action Theory	1	Puccio et al [90]

#### Were Behavior Change Theories Used Extensively?

Of the 16 eHealth systems, 9 were grounded in theory. However, the extent of theory utilization (the extent to which interventions address particular theory-relevant constructs [65]) varies substantially. Four interventions were supported by more than 1 theory. Of these, 1 (Hightow-Weidman et al [93]) was guided by 3 theories, whereas 3 interventions (Tanner et al [79,80], Spratt et al [82], and Shegog et al [83]) combined 2 theories. Table 3 provides an overview of the specific theories used per intervention. The remaining 7 interventions were not grounded in theory. While 3 of them mentioned a particular theory and its constructs in relation to adherence, they were not utilizing it to inform the design of the intervention. The remaining 4 did not refer to theory at all.

Based on the degree of application of theories in designing interventions, that is, based on the TCS [65] (also see Multimedia Appendix 3), we grouped studies into 4 usage categories as stated in the “Methods” section: no, low, medium, and high (Table 4). As much as 7 of 16 interventions have no use of theory, 5 have low theory usage, 1 used theory moderately, and 3 used theories extensively.

**Table 4:** The extent of theory usage of the included interventions, based on the Theory Coding Scheme [67].

Theory usage category			Theory Coding Scheme items		Interventions
**No**					
	No theory mentioned	Not applicable		Stankievich et al [81], Saberi et al [87], Dulli et al [100], Abiodun et al [101]	
	Mentioned theory	1		Puccio et al [90], Linnemayr et al [88,89], Dowshen et al [94,95]	
Low			2, 5, 8, 11		Spratt et al [82], Shegog et al [83], Belzer et al [84-86], Naar-King et al [91,92], Garofalo et al [96]
Medium			7, 9		Whiteley et al [77,78]
High			4, 6, 10		Tanner et al [79,80], Hightow-Weidman et al [93], Dworkin et al [97-99]

#### Were Developmental Changes Related to Adolescence Considered?

Given the importance of using suitable theoretical foundation for interventions that address adolescents (see the “Introduction” section), we explored whether the studies included in our review adequately address this aspect in how they report their work. Hence, we entirely examined them for information concerning the influence of developmental changes (biological, social, psychological) on ART adherence among adolescents. For example, we explored whether the studies considered the question of which theories could work better for adolescents (ie, which theory would provide better coverage of factors associated with developmental changes). Our findings suggest that none of the theory-informed eHealth interventions explicitly discussed theory selection from a developmental perspective: none of the studies provided a discussion of why or how the respective theories were appropriate for adolescents, suggesting lack of consideration of developmental changes. However, they mentioned previous use of those health theories in behavior change research including in adolescent populations (see Multimedia Appendix 2), although they did not include explicit reasoning for their choice. This may suggest that, in general, developmental changes related to adolescence are currently not included in the choice of theoretical foundation for interventions to increase ART adherence among adolescents in a transparent way, leaving room for future work that explicitly draws from suitable theory to achieve better outcomes as suggested by [102]. Additionally, we searched for evidence that developmental changes were accounted for in the intervention design—whether any specific design features were related to these changes—but no such explicit association was found.

### RQ2: Are eHealth Interventions Using Behavior Change Techniques?

#### Which Behavior Change Techniques Were Most Common?

Across the 16 eHealth systems reviewed, a total of 42 BCTs were identified (Multimedia Appendix 4). The most popular technique was “Prompting/cueing,” which was used by 11 interventions. The second most frequent technique was “Social support (unspecified),” used in 10 interventions. The third was “Problem solving,” used in 8 interventions. Next, “Monitoring of behavior by others without feedback” was used 7 times, and “Information about health consequences,” “Credible source,” “Social support (emotional),” and “Instructions on how to perform behavior” were each used 5 times. “Demonstration of the behavior” has been used 4 times and the remaining techniques appeared 3 times or less.

#### How Many BCTs Were Used Per Intervention?

The median number of BCTs used was 5. The intervention with the highest number of techniques was by Dworkin et al [97-99] utilizing 17 different ones. The lowest number of BCTs was found in Abiodun et al [101], using 1 technique. A complete list of details on how many techniques each intervention has applied is provided in Multimedia Appendix 4.

#### Are Behavior Change Techniques Linked to Behavior Change Theories?

Most theory-based studies (7/9: Shegog et al [83], Belzer et al [84-86], Garofalo et al [96], Whiteley et al [77,78], Tanner et al [79,80], Hightow-Weidman et al [93], Dworkin et al [97-99]) linked at least one intervention technique to 1 theory (TCS item 8) and at least one theory-relevant construct to an intervention technique (TCS item 11). However, only half (4/9: Whiteley et al [77,78], Tanner et al [79,80], Hightow-Weidman et al [93], Dworkin et al [97-99]) linked all theory-relevant constructs to at least one intervention technique (TCS item 10), and only 5 out of 9 (Whiteley et al [77,78], Tanner et al [79,80], Hightow-Weidman et al [93], Garofalo et al [96], Dworkin et al [97-99]) linked all intervention techniques to at least one theory-relevant construct (TCS item 7). Two (Shegog et al [83] and Naar-King et al [91,92]) studies used theory constructs to tailor intervention techniques to recipients (TCS item 6), but no study selected recipients for the intervention based on theory constructs (TCS item 4).

### RQ3. How Are eHealth Interventions Designed?

#### What Platforms Were Used to Implement eHealth?

Many of the systems were simplistic applications from a technical perspective developed on basic phones (ie, SMS text messaging [n=6] and phone calls [n=3]; Table 5). However, advanced applications designed for smartphones were also emerging (n= 3). These smartphone-based apps were designed in various forms—ordinary apps, serious games, and gamified apps. Already existing social media apps were also utilized. Electronic adherence monitoring devices (electronic medication containers that look like ordinary bottles or mobile phones, eg, WisePill) were also common, but mainly used in combination with other platforms and not as a standalone system. The primary purpose of these devices was to objectively measure adherence, except in 1 intervention (Spratt et al [82]) in which it was used to deliver reminders in the form of blinking lights and chime sounds. Other systems included web-based applications (desktop/laptop), remote videoconferencing, and desktop applications (Naar-King et al [91,92]). Such systems were, however, less frequent, each appearing just once.

**Table 5:** Type of systems in included studies.

System type	Frequency	Study
SMS text messaging	6	Stankievich et al [81], Spratt et al [82], Linnemayr et al [88,89], Dowshen et al [94,95], Garofalo et al [96], Abiodun et al [101]
Phone call	3	Spratt et al [82], Puccio et al [90], Belzer et al [84-86]
Electronic adherence monitoring device^a^	3	Whiteley et al [77,78], Spratt et al [82], Linnemayr et al [88,89]
Smartphone app	1	Dworkin et al [97-99]
Smartphone serious game	1	Whiteley et al [77,78]
Gamified smartphone app	1	Hightow-Weidman et al [93]
Social media	2	Tanner et al [79,80], Dulli et al [100]
Web-based application (desktop/laptop)	1	Shegog et al [83]
Videoconferencing	1	Saberi et al [87]
Desktop applications	1	Naar-King et al [91,92]

^a^Used in combination with others, not as a standalone intervention system.

#### Which Design Features Were Common?

Out of the 28 motivational design features proposed by Geuens et al [57], we found 24, with the most frequent features being “Reminders,” “Personalization,” and “General information”—appearing 11, 10, and 9 times, respectively (Multimedia Appendix 5). Information to educate adolescents about HIV and ART adherence was sometimes tailored to participants based on personal profiles—hence “Microtailoring.” “Instructions” on how to perform certain tasks such as taking medication and talking to providers were also detected. In some interventions, generic information and instructions were from health care expert sources (ie, “Expertise”). Asking patients to manually enter information (ie, “Logging”) about performance of their behavior (eg, whether they took medication or not) or outcome data (eg, viral load, CD4 count) was also common, although sometimes this was also done automatically, via “Tracking,” using electronic devices such as medication adherence monitoring devices.

#### How Many Design Features Were Used in the Different Platforms?

The median number of design features was 4. The intervention with the highest number of design features (n=15) was Hightow-Weidman et al [93], while the interventions with the lowest number of features (n=1) were Puccio et al [90] and Abiodun et al [101] (Multimedia Appendix 5).

### RQ4. What Are the Quality and Impact of the eHealth Interventions and How Do They Relate to Grounding in Theory?

#### Quality

We adopted Johnson et al’s [74] method to categorize papers on methodological quality, computing the quality of evidence score, ranging from 5 to 15. Papers with a rating 8 or below are categorized as “weak evidence,” 9-12 as “moderate evidence,” and 13 and above as “strong evidence.” Six interventions scored strong on quality of evidence, 4 moderate, and the remaining 7 weak (Table 6). The quality ratings only pertain to the strength of empirical evidence for outcome effects, and do not judge the overall quality of the studies.

#### Impact

As mentioned in the “Methods” section, outcome measures were coded following Information–Motivation–Behavioral Skills Model’s conceptualization of ART adherence behavior as a function of 3 mediators: information, motivation, and behavioral skills [47]. Therefore, the measures corresponding to these parameters are knowledge on HIV and ART, personal motivation and social motivation, and ART self-efficacy, respectively. Direct measures of adherence behavior are ART medication adherence and appointment adherence. Similarly, measures of biological outcomes include viral load and CD4 count. The number of measurements for each outcome, effect type, and quality of evidence is summarized in Table 6. Of the 16 eHealth systems, 10 reported positive effect on 1 or more of the outcomes measured, yet 7 of these also reported no effect on 1 or more of other outcomes. As much as 6 out of 16 reported purely no effect in all the outcomes measured.

**Table 6:** Impact of included interventions on Integrated Behavioral Model mediators of antiretroviral therapy adherence among adolescents and quality ratings.

Interventions	Theoretical grounding and motivational features			Quality and effectiveness^a^									
	Overall quality of theory integration (Table 4)	Behavior change techniques, n	Design features, n	HIV knowledge	Antiretroviral therapy (ART) knowledge	ART motivation (personal motivation)	Social support (social motivation)	ART self-efficacy	ART medication adherence	Medical appointment adherence	Viral load	CD4 count	Quality of evidence
Whiteley et al [77], [78]^d,e^	Medium	15	10	+	+	0	0	0	+		+		15
Tanner et al [79], [80]^e,f^	High	16	12							+	+		11
Hightow-Weidman et al [93]^e^	High	15	15	+	+			+					9
Dworkin et al [97]-[99]^e^	High	17	14	0				0	+				12
Stankievich et al [81]^g^	No	2	3								0		6
Spratt et al [82]^e,g^	Low	9	4						0			-	6
Saberi et al [87]^g^	No	7	2		0		0	0					6
Linnemayr et al [88], [89]^g^	No	3	2						0				13
Puccio et al [90]^g^	No	3	1						0		0		5
Naar-King et al [91], [92]^g^	Low	11	4						0		0		15
Dowshen et al [94], [95]^g^	No	2	3						+		0	0	8
Garofalo et al [96]^g^	Low	4	4						+		0		13
Shegog et al [83]^g^	Low	2	5	+	+	+		+					7
Belzer et al [84]-[86]^g^	Low	4	2			0		0	+		+		11
Dulli et al [100]^g^	No	6	4	+			0		0				13
Abiodun et al [101]^g^	No	1	1						0		+		14

^a^“+” means positive effect; “0” means no effect; “–” means negative effect.

^b^True for newly started ART; no significant effect on patients who stayed longer on ART.

^c^Higher usage of theory and motivational features, scoring at least medium and high once.

^d^Electronic monitoring alone (control) acts as a better intervention than additional signal and SMS text message reminders (intervention).

^e^Lower usage of theory and motivational features.

#### What Is the Relation Between Grounding in Theory and Impact of the Interventions?

The nature of this review precludes any firm conclusions. A closer look at Table 6 suggests mixed results; we cannot unambiguously conclude that more extensive grounding of design features in theory related to health behavior or behavior change is associated with better (significant) effectiveness on outcomes with good evidence quality (compare studies with footnotes c and e). However, the results suggest that 10/16 (Whiteley et al [77,78], Tanner et al [79,80], Hightow-Weidman et al [93], Dworkin et al [97-99], Dowshen et al [94,95], Garofalo et al [96], Shegog et al [83], Belzer et al [84-86], Dulli et al [100], Abiodun et al [101]) of the included eHealth apps report a positive impact on ART adherence or on its mediators.

## Discussion

### Summary

This systematic review examined theory usage, integration of BCTs, and motivational design features and technology platforms used in existing eHealth ART adherence interventions for adolescents, and how these aspects relate to the quality and impact of interventions. Generally, we found the impact of eHealth systems on adolescent ART adherence and its mediators promising. Moreover, most included systems attempted rooting eHealth interventions in theoretical frameworks. Nevertheless, we found a gap between the discussion of theories to root an intervention and the actual application of those theories in terms of system design. Additionally, we only came across few systems that contain a considerable number of BCTs and motivational design features. Instead, elementary designs characterize current systems. In the following paragraphs, we detail the main findings and relate them to existing theoretical and empirical work.

### Principal Findings

#### Are eHealth Interventions Grounded in Health Behavior Change Theory?

The review shows that current eHealth systems to improve adolescents’ ART adherence refer to theory only lightly. Of the 16 included eHealth systems, only 4 interventions show extensive to moderate usage of theory, while the remaining have low or no usage (Table 4). This finding contrasts with the studies that have argued that grounding the design of eHealth systems in theory is associated with increased effectiveness [40-42]. In the context of HIV in particular, designing eHealth systems based on behavioral theory is associated with efficacy in improving adherence to HIV medication [30]. This is attributed to the fact that health behavior theories provide a comprehensive understanding of ART adherence behavior and its determinant factors, to inform the design of the intervention [44,103]. Theory underutilization may result in a limited understanding of the different moderators of ART adherence among adolescents, leaving several factors unaddressed by the intervention, which in turn reduces effectiveness [104].

Moreover, our findings indicate that the context of developmental changes and their impact on ART adherence are not explicitly addressed in the included studies. This is somewhat surprising as prior research has shown that factors associated with developmental changes affect adolescents’ health including medication adherence [105]. Adolescence presents a specific developmental stage marked by profound biological, psychological, and social changes [106-111] that may affect ART adherence behavior [20]. For instance, we did not find explicit mention of how the ongoing development of cognitive capacity [17], oppositional behavior associated with becoming autonomous [21], and conformity to peer pressure [22] and the synergy it may have with HIV stigma [112] can affect ART adherence among teenagers. Being informed of these developmental theory concepts might be useful in the choice of appropriate BCTs and design features. However, the absence of explicit referral to developmental theory does not automatically imply that interventions are not (implicitly) informed by developmental theories.

#### Are Behavior Change Techniques Guided by Theory?

Almost half of the interventions applied only 4 or less BCTs, which may render them less effective in delivering intervention content: prior research on BCTs and health behaviors, (eg, [40-42,62]) has indicated that the number of BCTs applied in an intervention influences its effect, that is, interventions that employ more BCTs were found to have a larger effect on behavior than those that apply fewer BCTs [41]. This relatively low number of BCTs usage might be the result of a less comprehensive conceptualization of adherence and its determinants, or a less comprehensive conceptualization of adolescents’ developmental changes and their impact on ART adherence. For example, an intervention narrowing adherence to “consuming medication,” and focusing primarily on “forgetfulness” as main barrier, might end up employing the “prompts/cues” BCT only; “reminders” as the main technique can be seen in [90,94]. A deeper look through the perspective of developmental changes (ie, a reflection on why adolescents forget) might perhaps suggest another solution; for example, building adolescents’ skills on how to better integrate medications into daily life. In terms of breadth (covering a broad range of factors), reviews on barriers of adolescent’s ART adherence list many factors complicating adherence [9-11] that eHealth systems need to address. Moreover, we found that BCTs which might be relevant for adolescents such as demonstration of behavior (modeling), peer comparison, or incentivization were scarce.

#### What Are the Prevalent Platforms and Designed Features?

We found that most included systems were limited to SMS text messaging or phone calling. Compared with more advanced information and communications technology systems, SMS text messaging and phone calls offer the advantage of being cheap to deliver. However, such systems also limit the implementation of more sophisticated features. For example, it is challenging to deliver intervention content enriched with engaging audiovisual content. SMS text messages alone might not be sufficiently engaging to adolescents, which could be a possible explanation for researchers reporting noneffectiveness [88,113,114]. A previous work noted that for adolescents “texts that say the same thing are boring” [77], and the authors recommended dynamically changing the content of the message so that adolescents do not get tired of reading the same messages [89].

As for the designed features, “reminders (notifications)” and “personalization (adapting color schemes and skins)” were the most frequently applied followed by displaying “general information.” The remaining features were scarcely used. Providing reminders for medication, enabling customization on system features, and educating adolescents about HIV and ART are still appropriate. However, interventions could be more effective if they additionally include “social” features such as principles grouped under “reward and incentives” and “social interactions” categories [57].

#### What Are the Quality and Impact of the eHealth Interventions?

Of the 16 eHealth systems, 10 (Whiteley et al [77,78], Tanner et al [79,80], Hightow-Weidman et al [93], Dworkin et al [97-99], Dowshen et al [94,95], Garofalo et al [96], Shegog et al [83], Belzer et al [84-86], Dulli et al [100], Abiodun et al [101]) reported positive effect on 1 or more of the outcomes measured, yet 7 (Whiteley et al [77,78], Dworkin et al [97-99], Dowshen et al [94,95], Garofalo et al [96], Belzer et al [84-86], Dulli et al [100], Abiodun et al [101])out of these 10 also reported no effect on 1 or more of other outcomes. Six out of 16 (Stankievich et al [81], Spratt et al [82], Saberi et al [87], Linnemayr et al [88,89], Puccio et al [90], Naar-King et al [91,92]) reported purely no effect in all the outcomes measured. Overall, while evidence is mixed, the impact of these systems on ART adherence and its mediators has a positive trend. However, the nature of this review and diversity of eHealth studies preclude any firm conclusions based on our study findings (Table 6). Considering the relatively small number of digital interventions identified in this review and the mixed evidence on its impact, more digital interventions for adolescents’ HIV self-management should be evaluated to come to firm conclusions.

### Implications and Recommendations

#### Overview

A clear understanding of theoretical insights enables designers to translate various BCTs into features appropriate to this specific audience; the way BCTs are implemented and presented to users matters in respect to their effectiveness. Moreover, implementation sophistication on top of applying relevant intervention content is paramount. In the paragraphs below, we detail the recommendations derived from this review.

#### Ground Interventions in Theories and Methods Tailored to Adolescents

Deciding on a particular theory is challenging for eHealth designers as a plethora of health theories exist, and there is not one theory specifically developed for adolescents. In this respect, protocols for developing behavioral interventions such as the IM (intervention mapping) [115] and capability, opportunity, motivation, behavior frameworks [116,117] may be of interest. According to IM, one should first consult existing literature to list reported causes of the target health problem (in this case, barriers/facilitators of ART adherence among adolescents). Next, concepts from this list should be linked to theoretical constructs (ie, the theory with better coverage of this list could be a good candidate). Moreover, use of a combination of multiple theories (such as Integrated Behavioral Model or any customized combination) might be considered. Complementing this approach, the practical study of the actual target group (eg, user-centered methods such as participatory design [118,119] with adolescents) might also help identify the most relevant determinants (eg, [120]) on which an intervention can focus [121].

#### Selection of Behavior Change Techniques Tailored to Adolescents

Incentivization holds particular potential to improve adherence to HIV care [122-125] among adolescents. Moreover, as peer pressure among teen population is high [19], implementing social-based techniques involving peer role modeling and comparisons among peers might be effective. In this respect, Hightow-Weidman et al [93] reported that participants enjoyed “social-media-like” discussion boards that prompt peers to daily discuss on HIV matters and share experiences. However, any use of social media with adolescents should be done with utmost care, discussed further below.

Additionally, methods that help adolescents analyze barriers they face, and help them generate strategies to overcome these issues might be crucial. This is already addressed by some interventions that seek to develop problem-solving skills, for example, phone conversations with a professional adherence counselor [87] or a similar discussion with a humanlike character animated in computer software [91].

#### Implement Sophisticated Features With More Advanced Technology Platforms

More advanced and engaging design features such as gamification and social connections are needed in eHealth systems. Although evidence on the efficacy of gamification specific to adolescents’ ART adherence is not synthesized yet, literature on gamification and adherence indicated its potential [126,127] and positive influence on health behavior change in general [74,128-132]. First, as mentioned above, adolescents might be more attracted to immediate rewards [17] (eg, points) and incentives associated with adherence achievements (eg, badges) than foreseeing future long-term health consequences. In addition to material rewards, virtual rewards in the form of gamification elements such as points, levels, leaderboards, and easter eggs might be interesting. Additionally, as adolescents are heavily influenced by peer pressure [13], integrating “social interaction” principles [57] might be important. Allowing adolescents to link and share via social media platforms, connect with others in a similar condition and demonstrating their success (social identification), know how other peers are performing (social comparison), compete or cooperate with peers on social interaction environments could be useful. However, we acknowledge that given the specific developmental characteristics of this age group and the dangers of issues related to HIV stigma (eg, concerns similar to privacy unraveling [133,134]), any such designed features should be stigma sensitive and introduced with the greatest care.

Finally, to enable using more advanced features, implementations such as desktop applications, mobile apps, games, or including gamification elements and stigma-sensitive social media–like features might be more appropriate to engage teenagers. As such applications are emerging, implementations in these platforms are warranted in future interventions.

### Limitations and Future Work

As per our knowledge, this is the first systematic review to examine theory usage, BCTs, motivational design features, and technology platforms used in existing eHealth ART adherence interventions for adolescents. To investigate our main points, we used established coding frameworks and taxonomies. Nevertheless, this study comes with its limitations. First, this review focused primarily on exploring existing eHealth ART interventions for adolescents. There is a need for a more rigorous study of what best promotes ART adherence among adolescents, that is, looking at the future. Second, although established coding taxonomies were applied, and coders were trained, coding BCTs and design principles remained challenging due to variations in intervention content descriptions. Templates for reporting eHealth interventions for behavior change might be useful here. Finally, the findings in this review depended only on explicit referral to theories used to ground intervention and design. We must acknowledge that this methodological approach does not allow us to conclude that interventions and designs are not tailored toward adolescents if done implicitly. It may be that interventions are still informed by knowledge on adolescents’ developmental changes, but simply without explicit mentioning, for example, through the embodied knowledge of experts contributing to the design. Hence, future work may address this by including prespecified developmentally appropriate BCTs and incorporating these in the systematic review of eHealth interventions for ART adherence among adolescents. However, it should be noted here that, for the sake of reproducibility, design knowledge should be transparently communicated as a good practice.

### Conclusions

In this review, a total of 16 eHealth interventions targeting adolescents’ ART adherence were included. Overall, the impact of these systems on ART adherence and its mediators is promising, but evidence remains mixed. We observed mostly simple applications (ie, for SMS text messages and phone calls); however, advanced smartphone apps are emerging. Moreover, most interventions applied only a limited number of BCTs and designed features. While 9/16 systems were grounded in theory, overall, health theories were utilized sparsely. Moreover, we observed a dearth of approaches addressing the specific developmental changes related to adolescence and their implications for intervention design and responsiveness to BCTs. In summary, we suggest that eHealth interventions, as well as the design of specific delivery platforms, should include health theories that are appropriate to adolescents’ development, and implement features that cater to this age group.

## Appendix

Multimedia Appendix 1 Search terms structured as per the syntax of each database.

Multimedia Appendix 2 Characteristics of included studies.

Multimedia Appendix 3 Use of theory in the included interventions from the Theory Coding Scheme (TCS).

Multimedia Appendix 4 Presence of behavior change techniques (BCTs) from BCT taxonomy version 1 in the included interventions.

Multimedia Appendix 5 Presence of design principles in the included interventions from lenses of motivational principles.

## Abbreviations

ART: antiretroviral therapy
BCT: behavior change technique
TCS: Theory Coding Scheme
