# Supplementary material for: Behavioral Theories and Motivational Features Underlying eHealth Interventions for Adolescent Antiretroviral Adherence: Systematic Review
Source: JMIR Mhealth Uhealth. 2021 Dec 10;9(12):e25129. doi: 10.2196/25129 (PMC8709919; doi:10.2196/25129)
Supplement: Multimedia Appendix 1 [file mhealth_v9i12e25129_app1.docx]

| **Database** | **Query** |
| --- | --- |
|  |  |
| IEEE Xplore | (HIV OR "Human Immunodeficiency Virus" OR "HIV/AIDS" OR "Acquired Immunodeficiency Syndrome" OR "HIV-positive" OR "HIV.PLS." OR "living with HIV") AND (adolescent OR teen* OR young OR youth) AND (ARV OR antiretroviral OR "Antiretroviral Therapy" OR "HIV treatment" OR "HIV care") AND ("eHealth" OR "e-health" OR "electronic health" OR "digital health" OR telemedicine OR "tele-medicine" OR technology OR "computer-based" OR "web" OR "web-based" OR Internet OR online OR "social media" OR "social networking" OR "mHealth" OR "m-health" OR "mobile health" OR "mobile phone" OR "cell phone" OR "cellular phone" OR smartphone OR "text message" OR SMS OR "short message service" OR "app" OR "application" OR game OR videogame OR gamif* OR "play") AND (adherence OR attrition OR dropout OR drop-out OR completers OR "lost to follow-up" OR withdrawal OR nonresponse OR non-response OR "completion" OR "did not complete" OR retention OR loss OR compliance OR concordance) |
|  |  |
| ACM | Keyword:(HIV "Human Immunodeficiency Virus" "HIV/AIDS" "Acquired Immunodeficiency Syndrome" "HIV-positive" "HIV+" "living with HIV") AND Abstract:(adolescent teen* young youth) AND Fulltext:(ARV antiretroviral "Antiretroviral Therapy" "HIV treatment" "HIV care") AND Abstract:("eHealth" "e-health" "electronic health" "digital health" telemedicine "tele-medicine" technology "computer-based" "web" "web-based" Internet online "social media" "social networking" "mHealth" "m-health" "mobile health" "mobile phone" "cell phone" "cellular phone" smartphone "text message" SMS "short message service" "app" "application" game videogame gamif* "play") AND Fulltext:(adherence attrition dropout drop-out completers "lost to follow-up" withdrawal nonresponse non-response "completion" "did not complete" retention loss compliance concordance) |
|  |  |
| PubMed | (((((HIV[Title/Abstract] OR "Human Immunodeficiency Virus"[Title/Abstract] OR "HIV/AIDS"[Title/Abstract] OR "Acquired Immunodeficiency Syndrome"[Title/Abstract] OR "HIV-positive"[Title/Abstract] OR "HIV+"[Title/Abstract] OR "living with HIV"[Title/Abstract])) AND (adolescent[Title/Abstract] OR teen*[Title/Abstract] OR young[Title/Abstract] OR youth[Title/Abstract])) AND (ARV[Title/Abstract] OR antiretroviral[Title/Abstract] OR "Antiretroviral Therapy"[Title/Abstract] OR "HIV treatment"[Title/Abstract] OR "HIV care"[Title/Abstract])) AND ("eHealth"[Title/Abstract] OR "e-health"[Title/Abstract] OR "electronic health"[Title/Abstract] OR "digital health"[Title/Abstract] OR telemedicine[Title/Abstract] OR "tele-medicine"[Title/Abstract] OR technology[Title/Abstract] OR "computer-based"[Title/Abstract] OR "web"[Title/Abstract] OR "web-based"[Title/Abstract] OR Internet[Title/Abstract] OR online[Title/Abstract] OR "social media"[Title/Abstract] OR "social networking"[Title/Abstract] OR "mHealth"[Title/Abstract] OR "m-health"[Title/Abstract] OR "mobile health"[Title/Abstract] OR "mobile phone"[Title/Abstract] OR "cell phone"[Title/Abstract] OR "cellular phone"[Title/Abstract] OR smartphone[Title/Abstract] OR "text message"[Title/Abstract] OR SMS[Title/Abstract] OR "short message service"[Title/Abstract] OR "app"[Title/Abstract] OR "application"[Title/Abstract] OR game[Title/Abstract] OR videogame[Title/Abstract] OR gamif*[Title/Abstract] OR "play"[Title/Abstract])) AND (adherence OR attrition OR dropout OR drop-out OR completers OR "lost to follow-up" OR withdrawal OR nonresponse OR non-response OR "completion" OR "did not complete" OR retention OR loss OR compliance OR concordance) Filters: from 2000 - 2019 |
| SCOPUS | TITLE-ABS-KEY ( hiv  OR  "Human Immunodeficiency Virus"  OR  "HIV/AIDS"  OR  "Acquired Immunodeficiency Syndrome"  OR  "HIV-positive"  OR  "HIV+"  OR  "living with HIV" )  AND  TITLE-ABS-KEY ( adolescent  OR  teen*  OR  young  OR  youth )  AND  TITLE-ABS-KEY ( arv  OR  antiretroviral  OR  "Antiretroviral Therapy"  OR  "HIV treatment"  OR  "HIV care" )  AND  TITLE-ABS-KEY ( "eHealth"  OR  "e-health"  OR  "electronic health"  OR  "digital health"  OR  telemedicine  OR  "tele-medicine"  OR  technology  OR  "computer-based"  OR  "web"  OR  "web-based"  OR  internet  OR  online  OR  "social media"  OR  "social networking"  OR  "mHealth"  OR  "m-health"  OR  "mobile health"  OR  "mobile phone"  OR  "cell phone"  OR  "cellular phone"  OR  smartphone  OR  "text message"  OR  sms  OR  "short message service"  OR  "app"  OR  "application"  OR  game  OR  videogame  OR  gamif*  OR  "play" )  AND  TITLE-ABS-KEY ( adherence  OR  attrition  OR  dropout  OR  drop-out  OR  completers  OR  "lost to follow-up"  OR  withdrawal  OR  nonresponse  OR  non-response  OR  "completion"  OR  "did not complete"  OR  retention  OR  loss  OR  compliance  OR  concordance )  AND  ( LIMIT-TO ( PUBYEAR ,  2019 )  OR  LIMIT-TO ( PUBYEAR ,  2018 )  OR  LIMIT-TO ( PUBYEAR ,  2017 )  OR  LIMIT-TO ( PUBYEAR ,  2016 )  OR  LIMIT-TO ( PUBYEAR ,  2015 )  OR  LIMIT-TO ( PUBYEAR ,  2014 )  OR  LIMIT-TO ( PUBYEAR ,  2013 )  OR  LIMIT-TO ( PUBYEAR ,  2012 )  OR  LIMIT-TO ( PUBYEAR ,  2011 )  OR  LIMIT-TO ( PUBYEAR ,  2010 )  OR  LIMIT-TO ( PUBYEAR ,  2009 )  OR  LIMIT-TO ( PUBYEAR ,  2008 )  OR  LIMIT-TO ( PUBYEAR ,  2007 )  OR  LIMIT-TO ( PUBYEAR ,  2006 )  OR  LIMIT-TO ( PUBYEAR ,  2005 )  OR  LIMIT-TO ( PUBYEAR ,  2004 )  OR  LIMIT-TO ( PUBYEAR ,  2003 )  OR  LIMIT-TO ( PUBYEAR ,  2002 )  OR  LIMIT-TO ( PUBYEAR ,  2000 ) )  AND  ( LIMIT-TO ( DOCTYPE ,  "ar" )  OR  LIMIT-TO ( DOCTYPE ,  "cp" ) )  AND  ( LIMIT-TO ( SRCTYPE ,  "j" ) )  AND  ( LIMIT-TO ( LANGUAGE ,  "English" ) ) |
|  |  |
| Web of Science | (TS=(HIV OR "Human Immunodeficiency Virus" OR "HIV/AIDS" OR "Acquired Immunodeficiency Syndrome" OR "HIV-positive" OR "HIV+" OR "living with HIV") AND TS=(adolescent OR teen* OR young OR youth) AND TS=(ARV OR antiretroviral OR "Antiretroviral Therapy" OR "HIV treatment" OR "HIV care") AND TS=("eHealth" OR "e-health" OR "electronic health" OR "digital health" OR telemedicine OR "tele-medicine" OR technology OR "computer-based" OR "web" OR "web-based" OR Internet OR online OR "social media" OR "social networking" OR "mHealth" OR "m-health" OR "mobile health" OR "mobile phone" OR "cell phone" OR "cellular phone" OR smartphone OR "text message" OR SMS OR "short message service" OR "app" OR "application" OR game OR videogame OR gamif* OR "play") AND TS=(adherence OR attrition OR dropout OR drop-out OR completers OR "lost to follow-up" OR withdrawal OR nonresponse OR non-response OR "completion" OR "did not complete" OR retention OR loss OR compliance OR concordance)) AND LANGUAGE: (English) AND DOCUMENT TYPES: (Article)  Indexes=SCI-EXPANDED, SSCI, A&HCI, CPCI-S, CPCI-SSH, ESCI  Timespan=2000-2019 |
|  |  |
| ScienceDirect | Title, abstract, keywords: (HIV OR "Human Immunodeficiency Virus") AND (adolescent OR youth) AND ("Antiretroviral Therapy" OR "HIV treatment") AND ("eHealth" OR "text message") AND (adherence) Year:2000-2019 |
|  |  |

Original search date: 4/25/19
