# Supplementary material for: Behavioral Theories and Motivational Features Underlying eHealth Interventions for Adolescent Antiretroviral Adherence: Systematic Review
Source: JMIR Mhealth Uhealth. 2021 Dec 10;9(12):e25129. doi: 10.2196/25129 (PMC8709919; doi:10.2196/25129)
Supplement: Multimedia Appendix 2 [file mhealth_v9i12e25129_app2.docx]

| **Study description** | **Sample; Study design** | **Theory-basis; justification for theory selection; consideration of adolescent psychology** | **Impact** |
| --- | --- | --- | --- |
| Whiteley et al [77,78] 2018, USA; An immersive, action-oriented iPhone game to improve adherence to ART medication | HIV+ youth on ART (14–26 yrs), n = 61, 79% male, 97% Black, 74% non-heterosexual; RCT, 16 weeks | IMB; previous use; No | Significant improvement on HIV knowledge, ART knowledge, medication adherence and viral suppression. Non-significant increase on social support. No effect on ART self-efficacy. Negative effect on ART motivation. |
| Tanner et al [79,80]. 2018, USA. A social media intervention utilizing Facebook,texting, and GPS-based mobile social and sexual networking applications to improve HIV-care engagement and health outcomes. | Young men who prefer sex with men & transgender women  living with HIV (16–34 yrs.), n=9, 79.1% African American, 13.2%  Latino; Pre-post, 12-months | SCT, ET; No; No | Significant increase in medical appointment adherence and significant decrease on viral load |
| Stankievich et al [81]. 2018, Argentina.  Short messages sent via a preferred mobile based social network (WhatsApp or text messaging or Facebook). Option of two-way (extended) communication and recipients can be patient, parent or both. | HIV+ children & young adults  (6-25 yrs.) on ART with suboptimal  adherence (VL ≥ 1000 copies/mL),  n=20, females 68%; Pre-post, 8-months | No; NA, No | Non-significant decrease on viral load. |
| Spratt et al [82]. 2017, USA. Cell phone based motivational interviewing, positive reinforcement text messages. Alert signals on the MedMinder (blinking light, loud chime). | HIV+ adolescents (12-20 yrs.) on ART, non-adherent with detectable HIV viral load; n=12, 86.7% female, 100% African American; RCT, 6-month. | MI, TTM; previous use; No | Non-significant decrease in medication adherence, and significant decrease in CD4 count |
| Shegog et al [83]. 2012, USA. Web application providing game-like activities (including animation, interactive activities, peer, and expert video) that aimed to motivate adherence, raise awareness about HIV and ART medicines, adherence barriers and skills to overcome them. | Perinatally infected HIV+ youth  (14-22 yrs.), n=10, 80% female, 80% Black; Pre-/post-study with  a brief intervention session. | SCT, MI; previous use; No | Significant improvement on HIV knowledge, ART knowledge, ART-motivation and ART self-efficacy. |
| Belzer et al [84-86]. Study 1: 2014, USA. Study 2: 2018, USA. Daily cell phone conversations (once or twice a day depending on regimen) with a healthcare provider around self-care and taking HIV medications. | YLWH (15-24 yrs.), n=37, 62% male,  70% African American; RCT, 1 year (6-months intervention & 6-months post intervention), intervention: cell phone support, control: usual care. Study 1 - compares adherence and viral control outcomes between groups;  study2 – analyses impact of cell phone support on psychosocial outcomes in intervention group | Stress, Appraisal, and Coping Theory;  previous use; No | Significant improvement on medication adherence and viral suppression, non-significant increase on ART motivation and ART self-efficacy. |
| Saberi et al [87]. 2013, USA.  Medication adherence counseling session via videoconferencing with graphics and visuals. | HIV+ African American youth (18–29 yrs.) on ART, n=14, 86% male, and baseline mean self-reported adherence of 89%; Pre-post study a brief counseling session (45 minutes long). | No; NA; No | Non-significant increase on ART knowledge, perceived social support, and ART self-efficacy |
| Linnemayr et al [88,89]. 2017, Uganda.  SMS texting system to support adolescent ART adherence. | HIV+ youth (15-22 yrs.), n=332,  familiar with SMS texting; RCT, 1 year; random allocation to 3 groups (1-to-1-to-1): a weekly SMS group (1-way), weekly SMS with response option (2-way), usual-care control group. | IMB; previous use; No | Non-significant increase on medication adherence |
| Puccio et al [90]. 2006, USA.  Phone call based DOT (Directly Observed Therapy), with a decreasing number of calls (daily calls for 1st 4-weeks, 5days per week for next 4-weeks, and 3 days per week for the last 4-weeks ) | HIV+ youth (16-24 yrs.), n=8, 7 females, only five completed the study; Pre-post, 6-months, multiple points measurement; 12 weeks with intervention, 12 weeks post intervention follow-up. | No; NA; No | Non-significant improvement on medication adherence and viral suppression |
| Naar-King et al [91,92]. 2013, USA.  A two-session computer-based brief MI intervention by a humanistic avatar character. | HIV+ youth (16-24 yrs.), newly starting ART, n=76, 80% men, 20% female, 58 % gay/lesbian; RCT, 6-months, intervention (n=36): a two-session computer-based MI intervention, control (n=40): a two-session computer-based nutrition and physical activity. | MI; previous use, No | Non-significant improvement on medication adherence and viral suppression |
| Hightow-Weidman et al [93]. 2018, USA. Smartphone app embedding social networking and fundamental game mechanics, such as challenges, points, and rewards. | HIV+ youth men who prefer sex with men (16-24), n=20, 95% (19/20) nonwhite; Pre-post, 4-week pilot trial. | SCT, NC, FBM; previous use, No | Significant increase on HIV knowledge, ART knowledge, and ART self-efficacy. |
| Dowshen et al [94,95]. 2012, USA. Daily SMS reminder message, followed by another follow-up message after 1-hour to confirm whether participants took, medication asking them to reply 1 if they took and 2 if they did not. | HIV+ youth (14–29 yrs.) on ART with documented poor adherence, n = 25, 92% male, 60% black, 84% infected through unprotected sex; Pre-post 6- months trail. | No; NA; No | Medication adherence: significant increase;  Viral suppression & CD4 count: non-significant increase |
| Garofalo et al [96]. 2016, USA. 2 SMS messages sent around the time of  each scheduled dose: one reminding medication, the other confirming whether medication was taken with a structured response ("yes" or "no"). A set of motivational or encouraging follow-up messages were randomized depending on their affirmative or negative response. | Poorly adherent HIV+ adolescents and young adults (16–29yrs.), n=105, 80% male, 74% Black, 82% behaviorally-infected; RCT with 1 year follow-up, randomized into - intervention: daily 2-way SMS,  control: usual care, after 6-months trial the groups crossed over. | SCT; previous use; No | Medication adherence: significant increase;  Viral load: no effect |
| Dworkin et al [97-99]. 2019, USA.  Smartphone app with a talking human-like (avatar) embodied agent providing information, motivation, and behavioral skills. App designed specifically for young African American men who prefer sex with men to promote HIV medication adherence and retention in care. | HIV+ young African American men who prefer sex with men (18–34 yrs.) on ART for at least 3 months, n=32(completed); Pre-post, 3-months. | IMB; No, No | Pill count adherence: increased significantly; Self-reported adherence: non-significant increase; HIV knowledge: differentiating HIV & AIDS decreased, knowing CD4 and viral load increased; Self-efficacy: no change |
| Dulli et al. [100]. 2020, Nigeria. A Social media-based support group (Facebook secret group) for youth living with HIV to promote adherence and retention. Interactive discussions on various topics, sharing experiences, and supporting each other. | Youth (15-24) living with HIV, on ART; RCT for 22 weeks, total enrolled n= 349 (intervention group n=177, control group n=172, randomized), completed n = 324. | No; NA, No | Retention in treatment: no effect;  HIV-related knowledge: significantly better in the intervention group;  ART adherence: no significant differences  Social support: no significant differences |
| Abiodun et al. [101]. 2020, Nigeria. SMS reminders to improve ART Adherence among adolescents living with HIV. | Medication-non-adherent adolescents ((15 - 19 yrs)) living with HIV; RCT for 1 yr, n(completed) =209 (intervention n=105, control n=104, randomized) | No; NA, No | Viral load count: significant improve; ART adherence: no effect |

IMB=Information, Motivation and Behavioral skills model; SCT=Social Cognitive Theory; ET= Empowerment Theory; MI= Motivational Interviewing; TTM= Transtheoretical Model; NC= Narrative Communication; FBM= Fogg Behavior Model.
