# Supplementary material for: Behavioral Theories and Motivational Features Underlying eHealth Interventions for Adolescent Antiretroviral Adherence: Systematic Review
Source: JMIR Mhealth Uhealth. 2021 Dec 10;9(12):e25129. doi: 10.2196/25129 (PMC8709919; doi:10.2196/25129)
Supplement: Multimedia Appendix 3 [file mhealth_v9i12e25129_app3.docx]

|  | Whitely et al [77,78] | Tanner et al [79,80] | Stankievich et al [81] | Spratt et al [82] | Shegog et al [83] | Belzer et al [84-86] | Saberi et al [87] | Linnemayr et al [88,89] | Puccio et al [90] | Naar-King et al [91,92] | Hightow-  Weidman et al [93] | Dowshen et al [94,95] | Garofalo et al [96] | Dworkin et al [97-99] | Dulli et al [100] | Aiodun et al [101] | Total |
| --- | --- | --- | --- | --- | --- | --- | --- | --- | --- | --- | --- | --- | --- | --- | --- | --- | --- |
|  |  |  |  |  |  |  |  |  |  |  |  |  |  |  |  |  |  |
| 1.Theory/model of behavior mentioned | 1 | 1 | 0 | 1 | 1 | 1 | 0 | 1 | 1 | 1 | 1 | 1 | 1 | 1 | 0 | 0 | 12 |
| 2. Targeted construct mentioned as a predictor of behavior | 1 | 1 | NA | 1 | 1 | 1 | NA | NA | NA | 1 | 1 | NA | 1 | 1 | NA | NA | 9 |
| 3. Intervention based on a single theory (Not a combination of theories or theory and predictors) | 1 | 0 | NA | 0 | 0 | 1 | NA | NA | NA | 1 | 0 | NA | 1 | 1 | NA | NA | 5 |
| 4. Theory/predictors used to select recipients for the intervention | 0 | 0 | 0 | 0 | 0 | 0 | 0 | 0 | 0 | 0 | 0 | 0 | 0 | 0 | 0 | 0 | 0 |
| 5. Theory/predictors used to select or develop intervention techniques | 1 | 1 | 0 | 1 | 1 | 1 | 0 | 0 | 0 | 1 | 1 | 0 | 1 | 1 | 0 | 0 | 9 |
| 6. Theory/predictors used to tailor intervention techniques to – recipients | 0 | 0 | 0 | 0 | 1 | 0 | 0 | 0 | 0 | 1 | 0 | 0 | 0 | 0 | 0 | 0 | 2 |
| 7. All intervention techniques are explicitly linked to at least one theory – relevant construct | 1 | 1 | 0 | 0 | 0 | 0 | 0 | 0 | 0 | 0 | 1 | 0 | 1 | 1 | 0 | 0 | 5 |
| 8. At least one, but not all, intervention techniques are explicitly linked to at least one theory-relevant construct/predictor | 1 | 1 | 0 | 0 | 1 | 1 | 0 | 0 | 0 | 0 | 1 | 0 | 1 | 1 | 0 | 0 | 7 |
| 9. A group of techniques are linked to a group of constructs or predictors | 0 | 0 | 0 | 0 | 0 | 0 | 0 | 0 | 0 | 0 | 1 | 0 | 0 | 1 | 0 | 0 | 2 |
| 10. All theory-relevant constructs/predictors are explicitly linked to at least one intervention technique | 1 | 1 | 0 | 0 | 0 | 0 | 0 | 0 | 0 | 0 | 1 | 0 | 0 | 1 | 0 | 0 | 4 |
| 11. At least one, but not all, theory-relevant constructs or predictors are explicitly linked to at least one intervention technique | 1 | 1 | 0 | 0 | 1 | 1 | 0 | 0 | 0 | 0 | 1 | 0 | 1 | 1 | 0 | 0 | 7 |
| 12a. Theory-relevant constructs or predictors are measured (At least one construct of theory (or predictor) mentioned in relation to the intervention is measured post-intervention) | 1 | 0 | 0 | 1 | 1 | 1 | 0 | 0 | 0 | 0 | 1 | 0 | 1 | 1 | 0 | 0 | 7 |
| **Overall score** | 9 | 7 | 0 | 4 | 7 | 7 | 0 | 1 | 1 | 5 | 9 | 1 | 8 | 10 | 0 | 0 |  |
